# Why administration of lethal drugs should not be the role of the doctor

**DOI:** 10.1136/jme-2024-110678

**Published:** 2025-05-02

**Authors:** Sally Barker, Zoë Fritz, Alexander Ruck Keene

**Affiliations:** 1 King’s College London, London, UK; 2 THIS Institute (The Healthcare Improvement Studies Institute), University of Cambridge School of Clinical Medicine, Cambridge, UK; 3 Acute Medicine, Cambridge University Hospitals NHS Foundation Trust, Cambridge, UK; 4 39 Essex Chambers, London, UK; 5 Dickson Poon School of Law, Kings College London, London, UK

**Keywords:** Euthanasia, Death, Health Personnel

## Abstract

The suitability of doctors as agents of assisting dying remains debated, although it is common in many jurisdictions, and forms part of the proposed assisted dying legislation for England and Wales.

We examine the established philosophical and legal role of doctors in England and Wales and compare it to the active role required of doctors under proposed assisted dying legislation. For clarity, we refer to the latter role as ‘assisted dying practitioner’ (ADP). We interrogate two common claims: that (a) the role of doctors and ADPs holds normative conceptual analogy by virtue of shared goals to uphold autonomy and reduce suffering and (b) the practical skills required of ADPs are specific and exclusive to a doctor’s training. Examination reveals that neither claim is justified and, despite both roles prioritising patient welfare, identifies significant conflict between their primary goals. The role of an ADP is philosophically and legally singular. To add a duty to the role of the doctor which is antithetical to their overall goal of cure and disease prevention disrupts the defining characteristic of being a doctor: this may have repercussions on who applies to medicine, how doctors are trained, interact with patients and are regulated. We do not advocate for or against assisted dying but propose that if it is to be taken forward in England and Wales, society would be more appropriately served by considering the role of an ADP as a novel profession whose goals, competencies, research base and regulation can be established independently.

## Introduction

 The Terminally Ill Adults (End of Life) Bill^[^^1^^]^,^1^ providing for the limited legalisation of assisted dying, has passed its first major Parliamentary hurdle in England and Wales. The Bill would both permit capacitous, terminally ill adults to request assistance in dying and establish a framework for the provision of such assistance. Doctors, statutorily known as ‘medical practitioners’,^1 2^ are required under the Bill to perform several functions: to assess eligibility, to prescribe the necessary means, and to assist in administration as required by the individual in question.^1^ They are being positioned as both gatekeepers and facilitators.

The involvement of doctors in this way is increasingly common in the Western world.^3^ However, in England and Wales, the provision of assisted dying is not intended as a form of medical treatment. This is not a situation where Parliament is being asked to regulate a proposal by the medical profession that the prescription of lethal drugs should be regarded as an appropriate treatment for terminal illness, nor is the Bill seeking to dictate to doctors that this is the case. Rather, the Bill represents a strictly legislative approach,^4^ in which access to assisted dying is ultimately determined by the state as a societally legitimated response to the importance placed on autonomy^5^ (‘personal self-governance’^6^). Correspondingly, individual doctors are not duty-bound to participate under the Bill,^1^ and a doctor’s refusal to facilitate an assisted death would have no bearing on their professional status.^1^ Considering the provision of assisted dying as a state-sanctioned service, as opposed to a medical one, it is, therefore, relevant to question on what grounds doctors are justifiably required at all. Assisted dying is supported in principle by many doctors,^7^ but individual specialties remain divided in their official stance,^8^ and the involvement of doctors has received long-standing academic debate.^9^,^13^

In this article, we examine what justifies the involvement of doctors in assisted dying in the medicolegal context of England and Wales. According to the version of the Bill before Parliament at the time of writing this article, there are several ways in which doctors are being asked to support individuals in their settled desire to have assisted dying. First, to listen to and understand the individual’s concerns and reasons for wanting assisted dying and to discuss any alternative solutions. Second, assessing the capacity of a patient to make a clear and settled view on the course of action that they have chosen. Third, providing expert evidence in terms of the individual’s condition, prognosis and treatments available to a third party. Lastly, actively assisting in the death itself by prescribing and providing the lethal drugs, and by being present up until the time of death. We agree that doctors are well placed to undertake the first three of these: the UK General Medical Council has one of the duties of a good doctor as to ‘listen to patients and work in partnership with them, supporting them to make informed decisions about their care’,^14^ and considering prognosis and possibilities at the end of life is an integral part of this; doctors frequently already provide expert advice on patients clinical conditions to insurance companies and driving agencies, among others, as the profession uniquely qualified to do so; capacity assessments are integral to the regular work of doctors, although many other professions (social workers and judges, for example) can also do this. It is the last element—of actively prescribing and providing lethal drugs and being present for the individual to take them—that we argue is antithetical to the role of the doctor, and that asking doctors to do this risks causing significant unintended societal effects. To argue our case, we provide a philosophical concept analysis of the established role of doctors, by comparison to the active role required of doctors in assisted dying under the Bill. For clarity, we refer to the latter as that of an ‘assisted dying practitioner’ (ADP). By directly comparing doctors and ADPs, we interrogate two common claims: (a) the role of doctors and ADPs holds normative conceptual analogy by virtue of shared goals to uphold autonomy and reduce suffering^15 16^ and (b) the practical skills required of ADPs are specific and exclusive to a doctor’s training.^15 17^ Our analysis reveals that, despite both roles prioritising patient welfare, neither claim is justified and identifies significant conflict between the primary goals of each role. We do not advocate for or against assisted dying but propose that, if it is to be taken forward in England and Wales, society would be more appropriately served by considering the role of an ADP as a novel profession for which the goals, competencies, research base and regulation can be established independently.

### The role and goal of a doctor

Doctors are statutorily defined as medical practitioners.^2^ As such, doctors are legally protected under the common law principle of the ‘medical exception’ to perform acts which cannot otherwise be legitimised by consent, a common example being surgical amputation. This is on the premise that the intervention in question has been ratified by the medical profession as ‘proper’ or ‘reasonable’ medical treatment and is performed by a doctor in accordance with their duty of care.^18^

Although lists of medicine’s aims and goals have evolved over time, the goals of healing, relieving suffering and preventing disease remain largely consistent.^19 20^ However, there has been recent discussion as to whether such intentions can be combined into a singular, ultimate goal that defines the profession as a whole.^21 22^ A prevailing thesis is that of Broadbent: he proposes that medicine has a single, ultimate goal of pathocentric cure, with cure defined as ‘*an intervention that is reasonably effective at altering a course of a disease for the better’*.^21^ Consistent with this, medical practice in England and Wales remains a biomedical, ‘evidence-based model’ in which medicine is primarily evaluated according to its ability to correct biological dysfunction.^21 23^ Broadbent incorporates disease prevention by saying that this can be considered accessory to the goal of cure on the grounds that the intended outcome of both goals is the removal of disease.^21 24 25^^ [2]^One objection to an ultimate goal of cure concerns the recognised doctors’ role of providing symptom relief and alleviating suffering. Broadbent argues in response that symptom relief is, at best, subservient to cure, noting that medicine may cause further suffering in the pursuit of cure.^21^ This notion is supported by the principles of domestic medical law: a doctor who intentionally withholds the option of cure in favour of symptom relief being likely culpable for an act of medical negligence.^26^ The relief of suffering can, in other cases, be encompassed within an ultimate goal of cure: as noted also by Varga,^27^ medicine often employs symptom relief in order to facilitate cure. This can be a direct approach, such as using anaesthesia to ensure a patient can tolerate a surgical intervention. This can also be indirect, in which relieving symptoms acts to prevent further biological dysfunction, such as providing antiemetics for nausea and thus maintaining oral intake, or by providing pain relief to aid sleep. In this sense, relieving the symptom can remain in keeping with the moderate definition of cure as there is either an improvement of existing biological dysfunction or, under Broadbent’s conceptualisation,^21^ an act of disease prevention. This approach is arguably reflected by the aim of the modern hospice movement to help patients ‘to live until you die*’*; as attributed to its founder, doctor Dame Cicely Saunders.^28^ As such, it is challenging to identify an example of symptom relief within legitimate medical practice that does not ‘alter the course of a disease for the better’ to at least some extent.

Varga advances an alternative ‘Autonomy Thesis’ to define the role of the doctor; the ultimate goal of medicine, when contextualised to the West, is not cure but instead to uphold respect for individual autonomy.^27^ Autonomy, alongside beneficence, is considered a key moral principle of Western healthcare ethics.^6^ An essential part of autonomy-respecting practice is that a doctor listens to a patient and ensures that what is important to that particular patient is explored and understood.^14 29^ However, there is wide consensus^30^,^32^ that such principles cannot be taken as self-justifying or else ‘*the meaning of ‘morality’ has been watered down to mean something akin to rules of play’*.^30^ Of particular importance in the context of assisted dying, it is clear that autonomy can determine the consequences of medicine to a certain extent*,* for example, by a patient refusing specific lines of treatment, but this is distinct from determining what is offered as medicine in the first instance. Patients are permitted to refuse treatment, but as recently restated by the Court of Appeal,^33^ they cannot demand a treatment which is not clinically appropriate.

It is undeniable that medicine often fails to cure. Varga gives the example of a patient with a ruptured brain aneurysm.^27^ He is symptom-free but is certain to die of his aneurysm within days. In such circumstances, where there is no possibility of cure (in any sense), a doctor’s role is limited to prognostication, emotional support and symptom relief according to the patient’s needs and wishes. Varga argues that such activities demonstrate that ‘*autonomy is a legitimate [medical] aim’*.^27^ However, as posed by Broadbent,^21 34^ an inability to cure does not negate cure as medicine’s ultimate goal but rather necessitates distinction between the goals and the ‘business’ or competencies of medicine—in other words, an act may fail to cure without necessarily being considered something other than medicine, as long as one’s business remains directed towards the goal in question. This is also effective in countering the common objection termed ‘the puzzle of ineffective medicine’^21^; the mystery of how and why the practice of medicine has persisted throughout history despite consistent failure to cure even in the moderate sense.^28^ Broadbent’s ‘Inquiry Thesis’^21^ proposes the key competencies of medicine to be those of understanding and prediction, both of which are evident in Varga’s case. Alternatively, Smart has recently argued that, when specified to a Western context, the business of medicine is more appropriately conceptualised as provision of the ‘best available’ treatment (the ‘Best Available Intervention Thesis’),^25^ which necessarily includes the ability to determine when treatment would be futile. As such, both theses^21 25^ have different ideas about what the competencies of medicine are, but both agree that the competencies should be aiming towards a goal of cure.

### The role and goal of an ADP

In the medicolegal context of England and Wales, the role or practice of an ADP is novel and thus can only, at present, be inferred from the terms of the Bill itself. We have defined the role of an ADP as someone who prescribes and provides lethal drugs, assists the individual to take them, remains to support the individual until the time of death, and supports the bereaved. The procedure as a whole is described to have ‘failed’^1^ if death does not occur. As such, the ultimate goal of an ADP must encompass the facilitation of death. It is also reasonable to presume that an assisted death is intended to be a good death. Important features of a good death include relief from physical symptoms, autonomy in decision-making, good communication with care providers and ‘performance of cultural, religious and other spiritual rituals’.^35^ With regard to assisted dying, themes of relieving suffering and upholding autonomy have been consistently recognised as key regulatory goals within the legislative approach.^36 37^ Shared characteristics between ‘a good death’ and an assisted one were also highlighted within the recent House of Commons inquiry: an assisted death being described as ‘where the person dying was cared for with compassion and high-quality care and provided with as much agency and choice as possible’.^17^ Therefore, we can describe the ultimate goal of an ADP as being to facilitate a good, assisted death.

To succeed in their goal, the ADP must have pharmacological, psychosocial and cultural expertise. Pharmacological expertise must encompass the provision and administration of both lethal drugs and licensed medication for symptom relief. Regarding lethal drugs, expertise can be gained by reviewing the evidence base and by personal experience of prescribing drugs and seeing their effects. The evidence base is currently sparse. Different jurisdictions use different drugs^3^ and have inconsistent methods of collecting data regarding time taken to die and symptoms experienced.^3 38^ From the limited evidence available, complication rates appear to be relatively high, for example, annual summary reports of assisted dying under the Death with Dignity Act in Oregon reveal that approximately one in nine patients experience some form of complication including difficulty in ingestion, seizures and regurgitation.^38^ It is also important to note that different individuals have different dying needs. For example, a recent scoping review of assisted dying practice across several different jurisdictions records several instances in which the death was described to have ‘happened too quickly’.^3^ Work is needed to form a robust evidence base; prospectively collecting more data to determine the effects of different combinations of drugs on people with different conditions, weights and comorbidities.

The recipient’s welfare must remain paramount. Terminal illness is often associated with symptoms of pain, breathlessness and anxiety. While some may argue that death itself is the primary method to relieve suffering,^16^ the goal of a good, assisted death necessitates ADP expertise of symptom management at every stage of the process. This would also include additional symptom relief measures if, for example, complications or new symptoms were to arise related to drug administration. To ensure a good death, the ADP must also be able to provide practical psychosocial and cultural support to the individual who has chosen to have assisted dying. According to the version of the Bill before Parliament at the time of writing this article,^1^ this would also necessitate the ability to assess the capacity of the individual, up to and including the event of drug administration. Practical support might also be perceived to include support for the bereaved, which indirectly also supports the dying individual who can be reassured that their loved ones are being looked after.

### A comparison of roles

The prevailing arguments in support of doctors as ADPs tend to fall into one of two categories: (a) the argument that the role of doctors and ADPs holds normative conceptual analogy by virtue of shared goals to uphold autonomy and reduce suffering^15 16^ and (b) the argument that doctors hold ‘exclusive expertise’ of skills required by ADPs in facilitating death.^15 17^ We do not think these arguments stand up to scrutiny.

**Figure 1:**
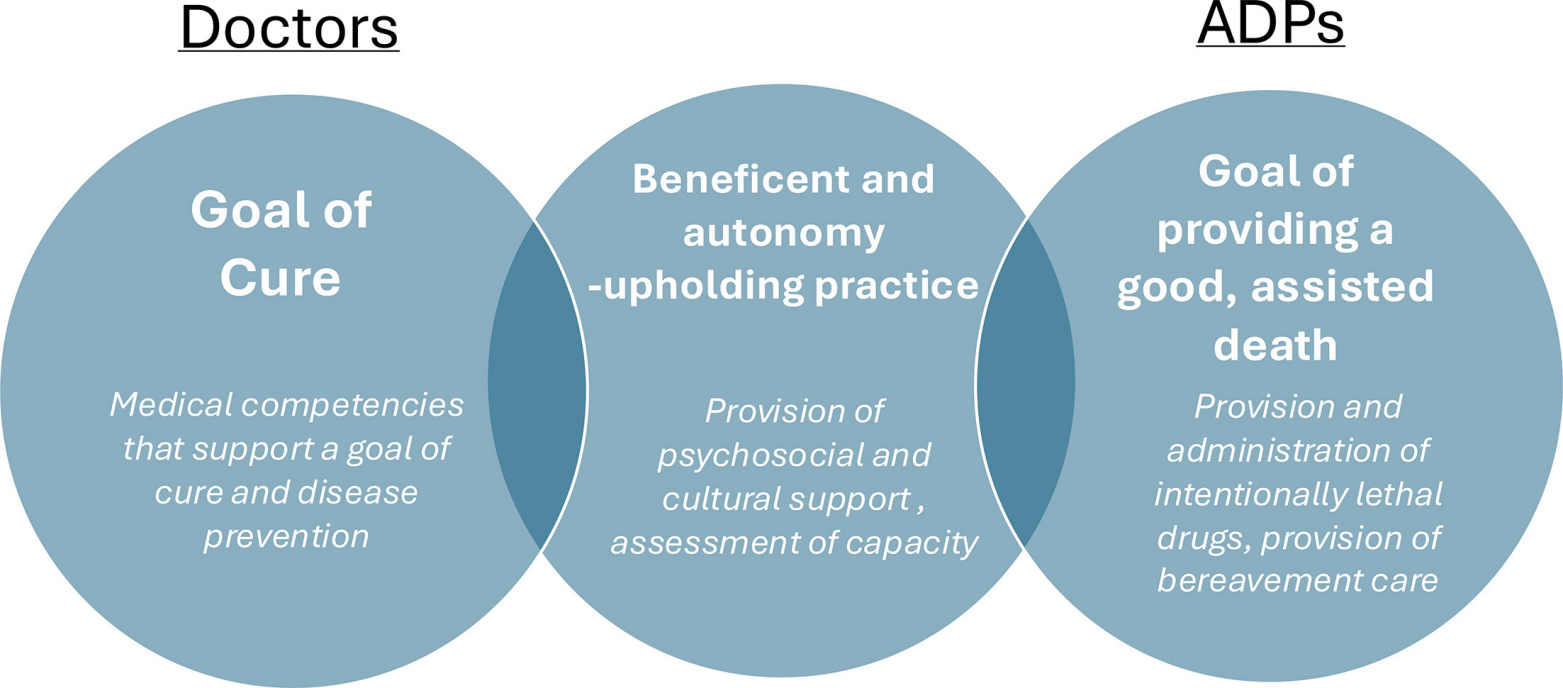
Comparing the goals and competencies of doctors and assisted dying practitioners (ADPs), respectively.

First, beneficence and autonomy-upholding acts are not under the exclusive remit of the medical profession. By both evolutionary^39^ and essentialist^32^ accounts, the guiding moral principles of medicine represent, at least to some extent, ‘a specification of general morality to the circumstances of medicine’.^30 40^ This has also been described by Beauchamp as ‘narrowing the scope of the norms, not by explaining what the general norms mean’.^40^ If assisted dying has been accepted within a general morality as a beneficent and autonomy-upholding, as is the premise of the Bill, deontological overlap with the morality of medicine is neither surprising, nor significant. What is important is the divergent goals of doctors and ADPS, with the former being driven by cure and preventing disease, and the latter being to facilitate a good, assisted death (figure 1). As remarked by a Swiss doctor participating in qualitative research on perspectives of assisted dying practice: ‘*how is it possible that at times we can be physicians and at times assistants in suicide?*’.^41^

Some may object to this conclusion with reference to palliative care, a medical specialty also renowned for its expertise in both symptom relief and care at the end of life. Historically, palliative care has also been claimed to be associated with hastened death, namely through provision of opioid medication and the practice of terminal sedation.^42^ We, alongside Schofield *et al*,^43^ maintain that this association is false. Critically, there is now a robust evidence base to show that routine opioid prescribing, according to national guidelines, does not shorten life. As such, ‘*we [can] reject the need for agnosticism and reaffirm that palliative opioid prescribing is safe’*.^43^ Meanwhile, terminal sedation, a practice which remains both unclearly defined and globally contentious, is not accepted as routine care in the UK.^43 44^ Therefore, neither opioid administration nor terminal sedation can be used to support the permissibility of death as a means to relieve suffering, in the context of medicine as practiced in England and Wales.

A second counter may accept our conclusion prima facie, but question why we cannot challenge the norm. Medical practice has evolved with time alongside scientific, technological and humanitarian progress. Of course, this will and should continue: we are not arguing ‘from tradition’ that doctors should not assist in death because they have never done so before, nor that the goals of medicine cannot shift or evolve with strong ethical arguments or societal need. Rather, given how widely cure as a primary goal of medicine is accepted, we argue that this goal should only change with explicit debate and strong ethical arguments, as opposed to it happening reactively as a consequence of another moral argument (the right to assisted dying), let alone a debate driven by Parliament, rather than by the medical profession itself.

Others may argue that vets have a long history of swapping between a goal of cure and a goal of active assistance in death, as need arises. However, the analogy between newly proposed legislation for assisted dying in humans and well-established animal euthanasia is flawed. The context of (and legislation for) animal euthanasia is very different to assisted dying in humans: animal euthanasia may be sought in response to animal suffering but may also be performed in the case where there is no one to care for the animal or, most commonly, as part of a larger process designed to provide animal food for humans to eat. It is legally permissible for anyone to perform ‘humane’ animal euthanasia^45^; vets are not required in order for euthanasia of animals to occur. However, humanely euthanising animals has been part of the duties of a veterinarian since the profession originated. McMillan has argued that comfort, not cure, is the primary goal of veterinarians and uses the duty to euthanise as an example of this: ‘*when all efforts to relieve discomfort fail to provide a reasonable amount of comfort for the animal, the decision to euthanatize often follows. Euthanasia is, perhaps, the clearest representation of our comfort objective; the reason for selecting this option is to eliminate discomfort in the only effective way left to us’*.^46^ Students undertaking studies in veterinary medicine are aware that euthanasia will form part of their duties, are taught how to do it, and it forms part of veterinary professional guidelines^47^; proving euthanasia is integral to, not counter to their goal.

Second, the expertise required to become an ADP is neither exclusive to doctors, nor, we may argue, always possessed by them; additional training will be needed for whoever takes on this role, and the assumption that it should be doctors because of similar competencies is misplaced. No medical practitioner in the UK currently has prior experience in the appropriate selection, prescription and administration of intentionally lethal drugs. Doctors are undoubtedly trained in the provision and administration of symptom-relieving medication, but nurses arguably have more experience in this, for example with ‘anticipatory’ or ‘just in case’ medication that is prescribed and dispensed in the community in anticipation of patients developing pain, breathlessness or anxiety, but can be administered by nurses as needed.^48^ Capacity assessment is a trainable skill shared by many professions. In complex circumstances, such as end-of-life decision-making, it is not uncommon for doctors to defer the assessment to a profession deemed to have more specialist knowledge, such as social workers, or to seek to obtain a determination from a judge of the Court of Protection. In an end-of-life context, doctors are trained in the provision of holistic medical care. However, non-medical roles, such as death doulas, arguably hold more relevant training and expertise in providing psychosocial and cultural support, as well as bereavement care,^49 50^ although this process—and the profession itself—is not yet regulated.^51^

Therefore, we suggest neither the arguments based on normative conceptual analogy nor exclusive expertise provide a compelling rationale for doctors being used as ADPs. Further, on a proper analysis, the roles are in direct tension: one working to improve biological function through cure, the other working to conclude it through death (figure 1).

### ADPS: a novel profession?

We suggest that requiring doctors to act as ADPs is not a neutral act. To add a duty to the role of the doctor which is antithetical to their overall goal of cure and disease prevention disrupts the defining characteristic of being a doctor and will fundamentally change the perception of what it is to be a doctor: this may have repercussions on who applies to do medicine, how doctors are trained, how they interact with patients, and how they are regulated.

To assist in death is philosophically and legally singular in the medicolegal context of England and Wales. Rather than straining both ethically and legally to extend the medical role to encompass working as an ADP, we suggest that it is better to recognise ADPs as a novel profession. Their goals can be clearly and unambiguously defined in legislation and amended in legislation to respond to developments. The competencies and training required for ADPs could be developed and evaluated. We anticipate several objections to our proposal, which we will now address.

First, there may be concerns that recruitment to the profession of ADP will be difficult, as attention to death has historically been considered taboo.^28^ However, the newly emerging role of death doulas has been welcomed and respected, and facilitating an assisted death is contextually associated with positive moral motivations,^4 17^ reinforced by evidence of strong societal support for legal change.^5^

A second objection may attempt to draw parallels between the arguments posed here and those put forward regarding a doctor’s role under the Abortion Act.^52^ Doctors have historically opposed participation in abortions on a range of grounds.^53^ However, fears that doctors would leave the profession on this basis remain unfounded, and rates of conscientious objection to abortion participation are perceived to have reduced over time. It is noteworthy that the intention of the Abortion Act ultimately remains to uphold the life and health of the pregnant individual^52^; this is directly in keeping with the concept of medicine’s ultimate goal as cure. Dissension regarding the permissibility of a doctor’s role under the Abortion Act depends to what extent the welfare of the fetus should be considered as a competing interest to that of the individual carrying the fetus. No such competing interests exist in decisions about assisted dying: it is the primary intent of ADPs in ending life that creates irreconcilable conflict with medical practice.

A third response could question why doctors should not consider being an ADP as simply an additional role alongside their traditional clinical duties. The Bill does not force or mandate individual doctors to participate—indeed, there is explicit protection for those who do not wish to do so.^1^ Hershenov argues that it is permissible for doctors to perform acts unconnected to medicine as long as they are neither in conflict with, nor at the neglect of, the practice of medicine.^12^ Medically trained and practising professionals frequently take on additional roles such as research, education and expert witness, all of which require additional training and carry ancillary goals. Individual agents may hold a differing set of intentions to those of their profession, financial remuneration being one example, and may take on additional roles with different goals outside their medical practice. The prescribing of homeopathic treatments by a registered medical practitioner is an example where the means of achieving cure is in conflict and may result in the neglect of the practice of medicine.^54^ In 2018, the High Court upheld that general practitioners (GPs) should not prescribe homeopathic treatments on the National Health Service.^55^ GPs are, however, still allowed to train in and practice homeopathy. A GP who was interested in homeopathy would only be permitted to offer the homeopathy as a homeopath, in a distinct practice. Similarly, a consultation could be had on whether doctors could train to become an ADP alongside their medical role. Instead of doctors having to ‘opt out’ by conscientiously objecting to contributing to assisted dying, as proposed in the current legislation, they could ‘opt in’ to have specialist training as an ADP. Consultation would include considering what limitations would be placed on this additional role, for example, limiting ADP practice to non-medical contexts and for individuals with whom they have no history of providing medical care. Meanwhile, others could also be trained and registered, for example nurses, vets and pharmacists.

## Conclusions

To summarise, we suggest that to require doctors, as doctors, to act as active agents of assisted dying is profoundly problematic. Claims of normative conceptual analogy are flawed, with the primary goals ultimately in conflict. The role of an ADP is philosophically and legally singular and should be recognised as such. While we do not argue either for or against the legalisation of assisted dying, we suggest that, if it is to be taken forward, society would be better served by considering the role of an ADP as a distinct, novel profession for which the goals, competencies, research base and regulation can be established independently.

## Data Availability

There are no data in our work.

## Footnotes

As introduced 11^th^ November 2024

The role of disease prevention with regards to the practice of medicine is an area of ongoing debate beyond the scope of this article. However, it is notable that Broadbent’s position is in keeping with Fuller’s parallel analysis of the role of preventive and curative medical circumstances in which disease is ‘probable’ to occur without intervention, and accepted by later critics of Broadbent’s overall thesis.
